# Analysis of Sigma-1 Receptor Antagonist BD1047 Effect on Upregulating Proteins in HIV-1-Infected Macrophages Exposed to Cocaine Using Quantitative Proteomics

**DOI:** 10.3390/biomedicines12091934

**Published:** 2024-08-23

**Authors:** Omar Vélez-López, Kelvin Carrasquillo-Carrión, Yadira M. Cantres-Rosario, Eraysy Machín-Martínez, Manuel E. Álvarez-Ríos, Abiel Roche-Lima, Eduardo L. Tosado-Rodríguez, Loyda M. Meléndez

**Affiliations:** 1 Department of Microbiology and Medical Zoology, University of Puerto Rico, Medical Sciences Campus, San Juan, PR 00936, USA; omar.velez3@upr.edu; 2 Integrated Informatics, Center for Collaborative Research in Health Disparities, University of Puerto Rico, Medical Sciences Campus, San Juan, PR 00934, USA; kelvin.carrasquillo@upr.edu (K.C.-C.); abiel.roche@upr.edu (A.R.-L.); eduardo.tosado@upr.edu (E.L.T.-R.); 3 Translational Proteomics, Center for Collaborative Research in Health Disparities, University of Puerto Rico, Medical Sciences Campus, San Juan, PR 00921, USA; yadira.cantres@upr.edu; 4 Department of Biology, University of Puerto Rico, Río Piedras Campus, San Juan, PR 00921, USA; eraysy.machin@upr.edu (E.M.-M.); manuel.alvarez3@upr.edu (M.E.Á.-R.)

**Keywords:** human immunodeficiency virus (HIV), cocaine, BD1047, monocyte-derived macrophages (MDM)

## Abstract

HIV-1 infects monocyte-derived macrophages (MDM) that migrate into the brain and secrete virus and neurotoxic molecules, including cathepsin B (CATB), causing cognitive dysfunction. Cocaine potentiates CATB secretion and neurotoxicity in HIV-infected MDM. Pretreatment with BD1047, a sigma-1 receptor antagonist, before cocaine exposure reduces HIV-1, CATB secretion, and neuronal apoptosis. We aimed to elucidate the intracellular pathways modulated by BD1047 in HIV-infected MDM exposed to cocaine. We hypothesized that the Sig1R antagonist BD1047, prior to cocaine, significantly deregulates proteins and pathways involved in HIV-1 replication and CATB secretion that lead to neurotoxicity. MDM culture lysates from HIV-1-infected women treated with BD1047 before cocaine were compared with untreated controls using TMT quantitative proteomics, bioinformatics, Lima statistics, and pathway analyses. Results demonstrate that pretreatment with BD1047 before cocaine dysregulated eighty (80) proteins when compared with the infected cocaine group. We found fifteen (15) proteins related to HIV-1 infection, CATB, and mitochondrial function. Upregulated proteins were related to oxidative phosphorylation (SLC25A-31), mitochondria (ATP5PD), ion transport (VDAC2–3), endoplasmic reticulum transport (PHB, TMED10, CANX), and cytoskeleton remodeling (TUB1A-C, ANXA1). BD1047 treatment protects HIV-1-infected MDM exposed to cocaine by upregulating proteins that reduce mitochondrial damage, ER transport, and exocytosis associated with CATB-induced neurotoxicity.

## 1. Introduction

Cocaine contributes to HIV-1 transmission and the development of neurocognitive disorders (HAND) by dysregulating immune cell function [1,2]. Cocaine increases viral progression and neurodegeneration [3,4,5]. Studies have demonstrated that cocaine use, along with HIV-1 infection, increases BBB dysfunction [6], viral replication [7], dysfunction of endothelial cells [8], infiltration of immune cells and inflammatory components into the brain, as well as neuronal and astrocyte degeneration [9,10,11,12]. In addition, cocaine promotes the secretion of neurotoxic factors from HIV-infected macrophages, including cathepsin B (CATB), a lysosomal cysteine protease that induces neuronal apoptosis in vitro and in vivo [5,13]. CATB concentrations increase in the plasma of HIV-1 seropositive patients who are cocaine abusers [10].

Cocaine binds to the Sig1R at physiological conditions of 2–7 µM [14]. Similarly, cocaine augments the secretion of extracellular vesicles (EVs) and infection-related molecules in T-cells, dendritic cells, and macrophages [15,16,17]. HIV-1-infected dendritic cells and macrophages show increased expression of Sig1R along with proinflammatory cytokines in their EVs when compared with T-cells [17]. We have previously reported that pharmacological modulation of Sig1R using the specific antagonist BD1047 before cocaine exposure reduces HIV-1 replication, CATB secretion, and neuronal apoptosis [1]. However, the pathways triggered by Sig1R interaction to decrease CATB secretion in HIV-1-infected macrophages are unknown. We hypothesize that the Sig1R antagonist BD1047, before cocaine exposure, significantly deregulates essential proteins involved in HIV-1 replication, CATB secretion, and neurotoxicity. This proteome dysregulation correlates with pathways that might include additional protein targets for the reduction of HIV-1 infection and CATB exocytosis.

Limited quantitative proteomics information is available that describes the combined effect of HIV-1 and cocaine in vitro using blood monocyte-derived macrophages (MDM). Proteomics analysis of HIV-1-infected MDM has been published using one-dimensional SDS polyacrylamide electrophoresis (1-DE) [18,19], surface-enhanced laser desorption ionization (SELDI) [20,21,22,23,24,25], matrix-assisted laser desorption/ionization-time of flight (MALDI-TOF) mass spectrometry [26], electrospray ionization liquid chromatography-tandem mass spectrometry (ESI LC/MS/MS) [26,27,28,29], and two-dimensional differential in-gel electrophoresis (2D DIGE) [29,30,31]. Other proteomics techniques for HIV-1 work include stable isotope labeling (SILAC) [32,33,34,35], isobaric tags for relative and absolute quantification [36,37], oxygen 18 isotope labeling [38,39], and label-free procedures [40,41,42,43,44]. We selected tandem mass tag (TMT) quantitative proteomics labeling for this study as it provides a platform for simultaneous analysis of many samples, reduces technical variability, and uses a high-throughput instrument with lower coefficients of variance [45,46]. However, to the best of our knowledge, there are no TMT proteomics studies in HIV-1-infected MDM exposed to cocaine using a pharmacological approach to modulate Sig1R. A similar approach was used in a study recently published by our group to determine the mechanisms of JWH-133 cannabinoid antagonist treatment in infected HIV-1 macrophages as a potential anti-inflammatory agent [47]. Since our group already demonstrated that BD1047 decreased HIV infection, CATB secretion, and neurotoxicity in infected MDM exposed to cocaine in vitro and in vivo, we thought of applying this quantitative proteomics approach to previously collected samples [1].

In this study, we present a quantitative and functional analysis of the relative protein abundance of HIV-1-infected MDM exposed to a combination of BD1047 and cocaine compared with unexposed controls. This work is important because it describes the protein networks affected by BD1047 treatment in HIV-1-infected MDM exposed to cocaine. The effect of BD1047 on HIV-1 infection, mitochondrial, and lysosomal dysfunction, as well as CATB secretion, trafficking, and processing, is also described. Together, these data suggest new roles in how the Sig1R antagonist BD1047 contributes to HIV-1 infection, CATB secretion in MDM, and its related neurotoxicity.

## 2. Materials and Methods

### 2.1. Isolation of MDM, HIV-1 ADA Infection, Cocaine Exposure, and Treatments with Sig1R Antagonist (BD1047)

Isolation of PBMC from peripheral blood of *n* = 11 women over 21 years, the MDM culture, and in vitro HIV-1 infection have been previously described in detail and summarized below [1]. In the previous study, Dr. Velez-López tested BD-1047 Sig1R antagonist and PRE-084 agonist. MDM-HIV and MDM-control lysates (*n* = 3) corresponding to BD-1047 treatment at the optimal concentration showing significant effect on decreasing CATB and neurotoxicity (10 micromolar) were selected for this study. The current work was approved by the University of Puerto Rico Institutional Regulatory Committee (IRB) (Protocol #0720416). Each donor willingly agreed to participate in the study and signed an informed consent. The privacy rights of human subjects were always observed by the Code of Ethics of the World Medical Association (Declaration of Helsinki of 1975).

Briefly, PBMCs previously isolated were cultured in T25 flasks (1 × 10^7^ cells/well) or six-well plates (5 × 10^6^ cells/well) in complete monocyte media (fetal bovine serum 10% (FBS), human serum 1%, RPMI, Penicillin/Streptomycin 1%) (Sigma Aldrich, St. Louis, MO, USA) and incubated at 37 °C in 5% CO_2_. Media was changed every 3 days, and after seven days, adherent cells were differentiated as ≥90% MDM as described [1,13]. After 7 days of differentiation, media was withdrawn, and MDM was infected in vitro with HIV-1ADA (0.1 MOI) for 18 to 24 h. After infection, uninfected or HIV-infected MDM was treated with media (non-treated group), cocaine (10 µM), Sig1R antagonist (BD1047 10 µM) alone, or Sig1R antagonist (BD1047 10 µM) one hour before cocaine treatment (10 µM) for 3, 6, 9 days post-infection (dpi) as described previously by cell viability and pharmacological assays. Supernatants were collected and stored at −80 °C. Levels of HIV-1 infection in MDM were assessed through p24 antigen levels in supernatants by ELISA as described previously [1].

### 2.2. Preparation of MDM Lysates and Protein Determination

Macrophages at 12 dpi were lysed and stored at −80 °C for proteomics studies. Briefly, MDM cultures were washed twice with sterile phosphate-buffered saline (PBS) and incubated with 100 µL of lysis buffer (5 mM Tris-HCl at pH 8.0, Triton X-100) containing 5 µL of protease inhibitor cocktail (AEBSF, Aprotinin, Bestatin, ethylenediaminetetraacetic acid (EDTA), and Leupeptin) as described for isolating the whole proteome in our previous studies [13]. Cells were incubated on ice for 30 min, and flasks were scraped for protein extraction. Each sample was carefully observed in the microscope each time after scraping to ensure proper MDM detaching from flasks. PBS buffer was used to wash each of the flasks several times. Samples were vortexed and centrifuged at 4 °C for 10 min at 1500 rpm.

Bicinchoninic acid (BCA) assay (DC Protein Assay, Bio-Rad, Hercules, CA, USA) was performed for total protein quantitation of MDM lysates according to the manufacturer’s instructions (Bio-Rad, La Jolla, CA, USA). Samples were assayed in technical replicates and read at 450 nm in a Varioskan Flash Spectral Reader (Thermo Fisher Scientific, Mount Prospect, IL, USA). Fifty (50) µg of total protein was aliquoted for future studies, and 20 µg of protein was further used for TMT analyses.

### 2.3. Preparation of Protein Samples for Tandem Mass Tag (TMT) Labeling

A total of twenty-four (24) protein samples (3 donors and 8 conditions) were used in a TMT 10-plex platform as described previously [48]. Briefly, for acetone precipitation, 50 µL of 10% sodium dodecyl sulfate (SDS) was added into a tube with 20 µg of protein from cell lysates, mixed, and heated for 15 min at 70 °C. Thereafter, cold acetone (~1 mL) was added to a final dilution of 15%, and samples were incubated overnight at −20 °C. The next day, samples were micro-centrifuged at 10,000× *g* for 10 min, and the supernatant was removed, followed by the addition of sample buffer (2× Laemmli Buffer + β-mercaptoethanol, Bio-Rad, USA).

Samples were heated at 70 °C for 10 min and run on a Mini-PROTEAN TGX Precast Gel 4–20% for 10 min at 200 volts. Gels were stained with Biosafe Coomassie G-250 stain and documented using a Chemi-Doc XRS+ (Bio-Rad, La Jolla, CA, USA) [48]. A representative gel image of the MDM protein extract for each of the treatments can be seen in Figure S1. Each of the gel lanes from the Coomassie-stained gels was cut out manually and diced into 1 mm^3^ cubes. Gel pieces were de-stained using a solution of 50% acetonitrile and 50 mM ammonium bicarbonate, alkylated with 10 mM iodoacetamide (IAA) in 50 mM ammonium bicarbonate for 45 min, and reduced using 25 mM dithiothreitol (DTT) in 50 mM ammonium bicarbonate for 30 min at 55 °C. Samples were then digested at 37 °C overnight with a grade-modified trypsin solution in 50 mM ammonium bicarbonate (Promega, Madison, WI, USA). Peptides were extracted from the gel pieces first using 150 µL of a mixture of 50% acetonitrile and 2.5% formic acid in water, then 150 µL of 100% acetonitrile, and then dried.

### 2.4. TMT Labeling

**Figure 1:**
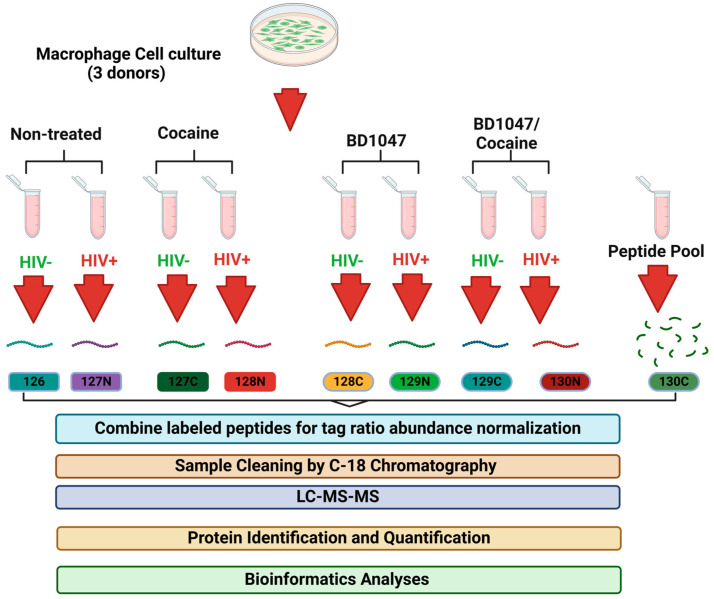
Experimental Design. Lysates from cultured human macrophages (MDM) in different conditions (HIV-infected, cocaine-exposed BD1047 treatments, and their controls) were acetone precipitated and subjected to SDS-PAGE. Gel regions were reduced, alkylated, and digested with trypsin. Each sample type was labeled with a unique TMT reagent. Samples were combined, desalted, and subjected to LC-MS/MS using a Thermo Q Exactive instrument, (Thermo Fisher Scientific, Mount Prospect, IL, USA). Proteomics analysis was performed using Proteome Discoverer Proteome Discoverer version 2.2 (Thermo Fisher Scientific, Mount Prospect, IL, USA), Limma Software, and IPA (IPA^®^, IPA; Ingenuity Systems, Qiagen, Redwood City, CA, USA).

Eight protein samples (100 µL) from HIV-infected and uninfected MDM lysates and one internal control pool of all samples were labeled with TMT10 plex Mass Tag Labeling kits (Thermo Scientific, Mount Prospect, IL, USA). Samples included four treatments (HIV only, HIV + cocaine, HIV + BD1047, HIV + BD1047/cocaine, uninfected only, uninfected + cocaine, uninfected + BD1047, uninfected + BD1047/cocaine). This makes for a total of eight samples. Since samples from three donors were used, this corresponded to 24 technical replicates (Table S1). The final pool is commonly used in TMT analyses to control the abundances of peptides in all samples and to normalize volume for further mixing of labeled TMT peptides as described previously [47,48]. A total of three kits were used for the three donors. Dried peptides were reconstituted in 100 mM triethylammonium bicarbonate (TEAB) buffer and subsequently labeled with the TMT10-plex reagents described in Figure 1. The labeling was performed following the manufacturer’s instructions (Thermo Fisher Scientific, Mount Prospect, IL, USA). Briefly, TMT reagents were resuspended in 41 µL of anhydrous acetonitrile (99.9%), added to each respective sample, and incubated for one hour at room temperature. Finally, the TMT reaction was quenched using 5% hydroxylamine for 15 min. After quenching was completed, 5 µL of each of the labeled samples were mixed in a single tube. The mixture was diluted tenfold in buffer A (0.1% formic acid in HPLC water) to decrease the acetonitrile concentration down to 3%. This mixture was analyzed by LC-MS/MS as described in the following section to make volume corrections for the final sample pools. For ratio check, 2 µL of labeled samples were injected into the Q-Exactive Orbitrap mass spectrometer (Thermo Fisher Scientific, Mount Prospect, IL, USA) for determination of the relative abundance of each reporter ion. Proteome discoverer (2.5) program (Thermo Fisher Scientific, Mount Prospect, IL, USA) was used for setting the defined parameters for the ratio checked per each TMT tag and exported to Microsoft Excel Program 2007 (Redmond, WA, USA). After exporting reporting ion abundances into the Excel program, these were averaged per TMT tag. Then, normalization of each reporter ion abundance was done by the following equation: $\frac{average\;mean\;abundane\;of\;each\;TMT\;tag}{mean\;abundance\;of\;TMT\;tag\;with\;greatest\;value}$ × 100. The TMT tag with the greatest value was assigned a 100% abundance. For dilution factor calculation, the same approach was used following equation $\frac{normalized\;TMT\;label\;with\;lowest\;value}{normalized\;TMT\;label\;of\;each\;sample}$ × 100. The lesser normalized TMT label was assigned a dilution factor of one. After ratio checks, all the calculated volumes per sample tag were mixed, and combined samples were dried in a speed vacuum for one hour. Samples were then cleaned up using the Pierce^®^ C18 Spin Columns (Thermo Fisher Scientific, Mount Prospect, IL, USA). For this procedure, dried samples were reconstituted in a sample buffer (2% trifluoroacetic acid in 20% acetonitrile) and C18 columns were activated by adding 50% methanol and centrifugation at 1500× *g* for one minute, twice. Equilibration solution (0.5% trifluoroacetic acid in 5% acetonitrile) was added and centrifuged for 1500× *g* for one minute, twice. Samples were loaded into each column of resin and centrifuged at 1500× *g* for one minute, twice. Wash solution (0.5% trifluoroacetic acid in 5% acetonitrile) was added, centrifuged at 1500× *g* for one minute, and repeated thoroughly. The elution solution (70% acetonitrile) was added to the top of the resin beds. Samples were centrifuged at 1500× *g* for one minute. Finally, cleaned samples were dried in a speed vacuum and stored for mass spectrometry analyses.

### 2.5. Liquid Chromatography/Mass Spectrometry (LC-MS/MS) Protein Identification and Quantitative Analysis

Peptide separation was performed using an HPLC system (Easy nLC 1200) (Thermo Fisher Scientific, Mount Prospect, IL, USA). Peptides were loaded onto a Pico Chip H354 REPROSIL-Pur C18-AQ 3 µM 120 A (75 µm × 105 mm) chromatographic column (New Objective, Littleton, MA, USA). The separation was obtained using a total gradient time of 128 min running at a rate of 300 L/min as follows: 7–25% of 0.1% of formic acid in 80% acetonitrile (Buffer B) for 102 min, 25–60% of Buffer B for 20 min, and 60–95% for 6 min. Separated peptides were electro-sprayed into and analyzed using a Q-Exactive Plus mass spectrometer (Thermo Fisher Scientific, Mount Prospect, IL, USA). The instrument was operated in positive polarity mode and data-dependent mode. The MS1 (full scan) was measured over the range of 375 to 1400 *m*/*z* and at a resolution of 70,000. The MS2 (MS/MS) analysis was configured to select the ten most intense ions for HCD fragmentation, configured over the range of 200 to 2000 *m*/*z* at a resolution of 35,000. A dynamic exclusion parameter was set for 30.0 s with a repeat count of three.

MS/MS raw data files were searched against a forward and reverse human protein database from UniProt (CC-BY 4.0 version 2021) (Universal Protein Source) (www.uniprot.org). Protein identification was performed using Proteome Discoverer version 2.2 (Thermo Fisher Scientific, Mount Prospect, IL, USA) with a SEQUEST HT algorithm. The search parameters included trypsin as the enzyme for proteolysis, in which two missed cleavages were allowed with a minimal peptide length of 6 and a maximal length of 144. Peptide mass tolerances were set at 20 ppm for the precursor mass tolerance and at 0.02 Da for the fragment mass tolerance. Dynamic modifications included oxidation +15.995 Da (M). Static modifications included carbamidomethyl +57.021 Da (C), TMT 6plex +229.163 DA (any N-terminus, K). The false discovery rate was set at 0.01 (strict) and 0.05 (relaxed). Obtained raw protein files were exported from Proteome Discoverer software version 2.1 into a .xls format using Microsoft Excel Program 2016 (Redmond, WA, USA).

### 2.6. Statistics and Bioinformatics Analyses

Protein abundances were analyzed using the Bioconductor package R-Limma for statistical analysis [49]. Before statistical analysis with Limma software (version 3.60.4), missing protein abundances across all group comparisons were eliminated by applying Base R software (version 4.1.0) commands. A custom Python program (Phyton programming language Version 3.x, Phyton Software Foundation, 2023, www.phyton.org , Wilmington, DE, USA) was developed for cleaning and preparing the data into the input format into tables that consisted of accession protein number and its related abundance per TMT tag per donor per treatment required for the analysis. The Python program arranged these accession numbers and abundances into matrixes that were further used for Limma software.

To calculate their respective fold changes and *p*-values, a total of three different comparisons (experimental cases/controls) of protein abundances were analyzed with the Limma software. Single channel analyses included abundances among [HIV infected (127N) vs. uninfected (126)], [HIV infected + cocaine (128N) vs. HIV infected (127N]), and finally [HIV infected BD1047 + cocaine (129C)] vs. [HIV infected + cocaine (128N)] (Table S1). Information on deregulated design linear model groups of comparison as well as all proteins without significant *p*-values can be seen on Supporting Information Electronic Data deposited in PRIDE. For fold change calculations, the mean protein abundances of each experimental condition were divided by the mean protein abundance of their control $\frac{\left(Mean\;protein\;abundance\;experimental\right)}{(Mean\;protein\;abundance\;control)\;}$. Then, these matrixes were quantified based on linear models described by the functions of $E_{\left(y_{g}\right)=X\beta_{g}}$ where for each protein (g), we have a vector of protein expression values $\left(y_{g}\right)$ and a design matrix (X) that correlates three values to some coefficient of interest ($\beta_{g}$). Variation of samples was expressed as $var\left(y_{gj}\right)=\frac{\sigma_{g}^{2}}{w_{gj}}$. The Limma software package includes empirical Bayesian methods to obtain variance estimators. Since all the protein abundances fit under a Gaussian distribution, several Student’s paired *t*-tests were performed. Statistically significant proteins were obtained as a result, with fold change (FC) values greater or equal to the absolute value of the module of one point five and *p*-value lower or equal to 0.05 (i.e., FC ≥ |1.5| and *p*-value ≤ 0.05, 95% confidence). Proteins whose fold changes are under the fold changes pertaining to [−1.5 ≤ x ≤ 1.5] were considered deregulated; those with fold changes under x ≤ −1.5 were considered downregulated, while those with fold changes over x > 1.5 were considered upregulated.

We analyzed the dysregulated proteins in our study using several graphical representations, including volcano plots, heatmaps, and Venn diagrams. We compared three groups: HIV-infected vs. uninfected individuals, HIV-infected individuals with and without cocaine use, and HIV-infected individuals treated with BD1047 plus cocaine vs. those treated with cocaine alone. We generated Venn diagrams using Venny, an interactive tool that facilitates the comparison of lists through Venn diagrams [50]. To create the volcano plots, we utilized the VolcaNoseR web application, as described in its publication [51]. We used GraphPad Prism version 10.2.0 (GraphPad Software, Boston, MA, USA, www.graphpad.com) to visualize the fold changes across comparisons in heatmaps. This approach allowed us to identify unique and shared patterns of protein dysregulation across the three groups we studied.

### 2.7. Ingenuity Pathway Analyses (IPA) and Literature Review

The lists of 94 differentially expressed proteins were uploaded to Qiagen Ingenuity Pathway Analysis (IPA^®^, Ingenuity Systems, Qiagen, Redwood City, CA, USA) software for CORE analysis and molecular annotations using the Ingenuity Pathway Knowledge Base (version 21.0). The group comparisons uploaded to IPA were HIV+ vs. HIV−, HIV+ cocaine vs. HIV+, and HIV+ BD1047/cocaine vs. HIV+ cocaine. The analysis was performed to identify proteins associated with Lysosome and CATB network interactions; Lysosome and Mitochondria network interactions; and HIV Infection Protein-Protein Interaction for the group HIV+ cocaine vs. HIV+ and HIV+ BD1047/cocaine vs. HIV+ cocaine. The cutoff to consider significant proteins in the CORE analysis was based on a fold change ≥ |1.5| and *p*-value ≤ 0.05. The human model was considered the model organism for annotations. Further, protein selection was based on IPA nodes representation as well as primary literature. Deregulated proteins were chosen based on fold change differences and significant *p*-values (≤0.05) from three biological replicates (*n* = 3). Selection of candidate proteins for further studies was based on the following criteria: (1) Significant fold change differences and/or *p*-values were based on Limma analyses; (2) Ingenuity Pathway analyses relating each subjected topic of mitochondrial, lysosomal, and cathepsins; and (3) Literature-based function regarding their role with HIV-1 infection, lysosome, and mitochondrial instability, CATB trafficking, processing, or secretion.

## 3. Results and Discussion

### 3.1. Pretreatment with BD1047 Prior to Cocaine Reduces HIV-1 Infection, CATB Secretion in Infected MDM, and TMT Group Analyses

In previous studies, we reported that HIV-1-infected MDM exposed to cocaine showed decreased infection and CATB secretion after the addition of the Sig1R antagonist BD1047 [1]. According to the previous results, MDM from three donors was infected with HIV-1 in vitro, exposed to cocaine or cocaine with no virus, and pre-treated with BD1047. Infection, as measured by HIV-1 p24 antigen levels, increased to higher levels in the presence of cocaine [1]. The effect of cocaine on boosting HIV infection in MDM was also reported in another study [10]. We demonstrated that HIV-1-infected MDM without treatment averaged 5.0 × 10^4^ pg/mL, while those infected and exposed to cocaine increased further to 1.2 × 10^5^ pg/mL at 13 days post-infection. However, when HIV-infected MDM were treated with BD1047 before cocaine exposure, the HIV-1 p24 antigen decreased significantly when compared with the other groups (˂2.5 × 10^4^ pg/mL) [1]. Therefore, for this study, we selected 13-day MDM lysates stored at −80 °C from the previous study and analyzed by TMT quantitative proteomics and bioinformatics. The TMT tags for each of the treatments included the following three comparison groups: (1) HIV infected (127N) vs. uninfected (126), (2) HIV infected + cocaine (128N) vs. HIV infected (127N), (3) HIV infected + BD1047 + cocaine (130N) vs. HIV infected + cocaine (128N) (Figure 1). Since our hypothesis was to determine the signaling pathways of Sig1R antagonist BD1047 modulation of CATB secretion, HIV-1 infection, and lysosomal and mitochondrial network interactions, we focused our results on the third comparison group of HIV infected + BD1047 + cocaine with HIV infected + cocaine in the absence of BD1047. Nevertheless, a discussion of the intracellular pathways in HIV-1 infection and cocaine exposure is included (Figure 1).

### 3.2. Differentially Expressed Proteins in Macrophages Among Comparison Groups

**Figure 2:**
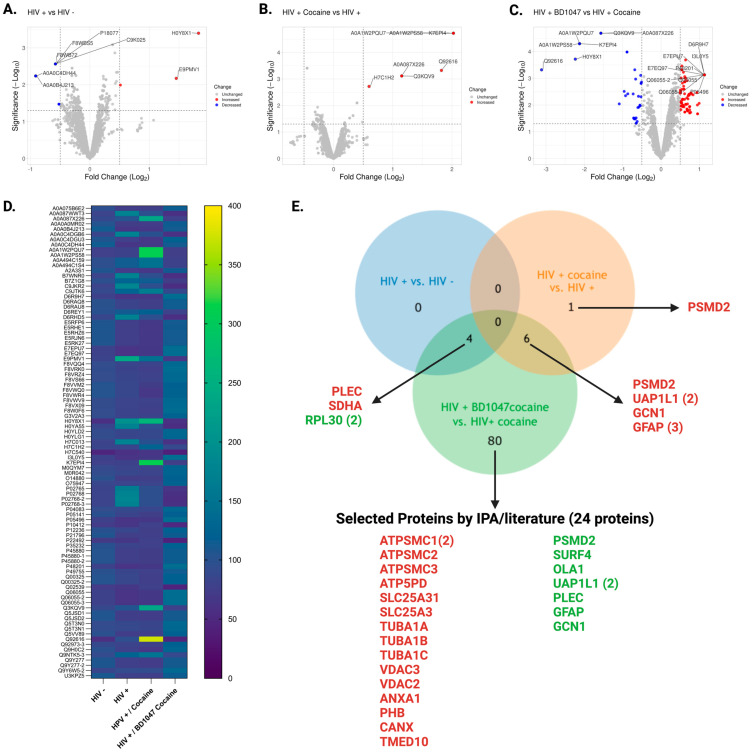
Differentially abundant proteins in MDM between the different comparison groups. (**A**) Volcano plot with the top significantly differentiated protein in HIV+ versus HIV−. (**B**) Volcano plot with the top significantly differentiated protein in HIV+ cocaine versus HIV+. (**C**) Volcano plot with the top significantly differentiated protein in HIV+ BD1047/HIV+ cocaine versus HIV+ cocaine. (**D**) Heat map on the top significant differentiated proteins based on FC = |1.5| and *p*-values ≤ 0.05. (**E**). Venn diagram the total number of differentially abundant proteins based on FC = |1.5| and *p*-values ≤ 0.05 that are unique or shared among groups. Proteins were selected for the BD047/cocaine group based on literature review and IPA analyses. The red color indicates upregulated protein, and green indicates downregulated protein.

After processing macrophage lysates from the three comparison groups for TMT, LC-MS/MS, and proteomics analyses, 5096 raw proteins were identified from the first donor, 4877 from the second donor, and 4939 from the third donor (raw data deposited in PRIDE). Thereafter, proteins were quantified based on the exclusion criteria of at least two unique peptides. The number of proteins per donor was reduced when exclusion criteria were applied to 1435 for the first donor, 1435 for the second, and 1444 for the third, respectively (Table S2). After considering the statistical parameters of fold change ≥ |1.5| and a *p*-value ≤ 0.05, we found that there are no unique proteins dysregulated by HIV-1 infection (HIV+ versus HIV−) groups. Two upregulated proteins (plectin, succinate dehydrogenase complex flavoprotein subunit) and two downregulated protein fragments (large ribosomal subunit protein eL30), for a total of four (4) proteins, are shared with the BD1047/cocaine group. These proteins contribute to cell structure, respirative oxidation, and protein synthesis (Table S2). When we compared the HIV + cocaine versus HIV+ group, we found seven (7) proteins that were upregulated and shared with the HIV + BD1047/cocaine group: UDP-N-acetylglucosamine pyrophosphorylase 1 like 1 (UAP1L1), stalled ribosome sensor GCN1 (GCN1), and three glial fibrillary acidic protein fragments (GFAP). BD1047/cocaine and cocaine groups are both dysregulated proteins related to protein synthesis, modification, and cell integrity. Dysregulation of only one protein, the proteasome 26S subunit ubiquitin receptor, non-ATPase 2 (H7C1H2) in the HIV + cocaine group further strengthens our observation of mechanisms to protect protein synthesis (Table S2). When comparing HIV+ BD1047/cocaine versus HIV+ cocaine groups by Limma analyses, a total of eighty (80) differentially abundant groups were identified. Of those differentially abundant proteins, sixty-four (*n* = 64) proteins were more abundant and twenty-nine (29) less abundant (Table S2). From those proteins, we selected twenty-four (*n* = 24) based on their significance for HIV-1 infection, mitochondrial dysfunction, lysosomal dysfunction, and cathepsin B. Sixteen (16) differentially more abundant (upregulated) and four (4) less abundant (downregulated) proteins have strong associations with mitochondrial dysfunction, lysosomal function, cathepsin B, and HIV infection. Newly identified proteins per group of analyses can be seen on the Venn diagram in Figure 2.

### 3.3. BD1047 and Cocaine Dysregulated Biologically Important Proteins That Are Related to Infection, Mitochondrial Function, ER Transport, and Cytoskeleton Remodeling in HIV-1-Infected MDM

#### 3.3.1. Differentially Abundant Proteins after HIV-1 Infection of MDM

HIV-1 infection in MDM (HIV+ versus HIV−) upregulates two proteins related to cell morphology, plectin (PLEC) and oxidative phosphorylation succinate dehydrogenase complex subunit A (SHAD), as well as two subunits of the large ribosomal subunit protein eL30 (RPL30) shared with the BD104/cocaine group (Table 1, Figure 2). Plectin is a protein complex associated with actin-binding intermediate filaments that associates with chemokine receptor X4 (CXCR4). Knockdown of plectin reduced CXCR4-tropic HIV-1 infection in MAGI (HeLa-CD4-LTR-Gal) cells [52]. Similarly, C-C chemokine type receptor 5 (CCR5), the major receptor for HIV-1 entry in MDM, increases plectin expression in monocytes [53]. Plectin expression is related to cytoskeletal arrangements induced by the virus after it enters the cells. On the other hand, upregulation of the succinate dehydrogenase complex subunit A (SDHA) is in accordance with previous studies indicating that the virus downregulates the cell glycolytic pathways and increases the tricarboxylic acid (TCA) proteins for further oxidative respiration as a mechanism for maintaining proviral reservoirs [54]. Similarly, large ribosomal subunit protein eL30 upregulation indicates a rescue mechanism in response to protein failure and the takeover of the virus of the protein machinery in the cell [55]. The sharing of dysregulated proteins by the BD1047/cocaine group shows that BD1047 pretreatment does not alienate key regulatory infectivity mechanisms independent of cocaine exposure in MDM. However, no unique proteins among the HIV+ versus HIV- group were found (Figure 2).

#### 3.3.2. Differentially Abundant Proteins in HIV-1-Infected MDM after Exposure to Cocaine

HIV-1-infected MDM exposed to cocaine, in comparison to HIV alone, revealed seven upregulated proteins in HIV-1-infected MDM (Table 2). These include the proteasome 26S subunit ubiquitin receptor, non-ATPase 2 (H7C1H2), two fragments of UDP-N-acetylglucosamine pyrophosphorylase 1 (UAP1L1), the stalled ribosome sensor GCN1 (GCN1), and three glial fibrillary acidic protein fragments (GFAP). Cocaine addiction has been reported to upregulate one unique protein named the proteasome 26S subunit ubiquitin receptor (H7C1H2) when compared with the infected group without the drug. It is fairly known that the virus hijacks the cell proteasome-ubiquitination pathways to produce its viral proteins. Specifically, HIV-1 hijacks the expression of the 26S proteasome complex through the use of ubiquitin proteins termed UPS [56]. Drugs of abuse, such as cocaine, further potentiate this action in many cells [57,58]. The 26S proteasome complex is responsible for tagging misfolded proteins and damaged proteins in the cell. These results suggest that both the virus and the drug of abuse synergistically collaborate for “protein dysregulation chaos” within the infected cell. Both infected MDM pretreated with BD1047 and those treated with cocaine-only groups shared six proteins after IPA analysis: two fragments of sugar addition in proteins to be degraded: UDP-N-acetylglucosamine pyrophosphorylase 1 like 1 (UAP1L1), protein synthesis-stalled ribosome sensor GCN1 (GCN1), and three fragments from cell cytoskeleton filaments glial fibrillary acidic protein (GFAP). As mentioned above, the virus potentiates the expression of proteins that might be used for its benefits. However, the upregulation of UAP1 proteins indicates a regulatory mechanism to increase the Interferon Type 1 response, a possible mechanism for reducing HIV-1 infection in MDM [59]. Similarly, upregulation of the stalled ribosome sensor (GCN1) indicates a way to reduce HIV-1 infection by limiting HIV-1 integrase and HIV-1 replication [60]. GCN1 forms a complex with another sensor, GCN2, and limits the binding of the HIV-1 integrase into the cell genome. Although this protein is increased by cocaine, it is further upregulated by the pretreatment with BD1047, which might explain some of the reducing effects of the antagonist prior to cocaine. Finally, glial fibrillary acidic protein (GFAP) is upregulated in the cocaine group but downregulated by BD1047 pretreatment. GFAP is an intermediate filament of astrocytes, and its expression is usually high in astrocytes, promoting astrogliosis and inflammation of the central nervous system in patients with HIV-1 dementia. Its expression is further increased by cocaine, and the addition of the antagonist indicates that these effects are abrogated [61].

#### 3.3.3. Findings of Differentially Abundant Proteins in HIV-1-Infected MDM Pretreated with BD1047 and Exposed to Cocaine

**Figure 3:**
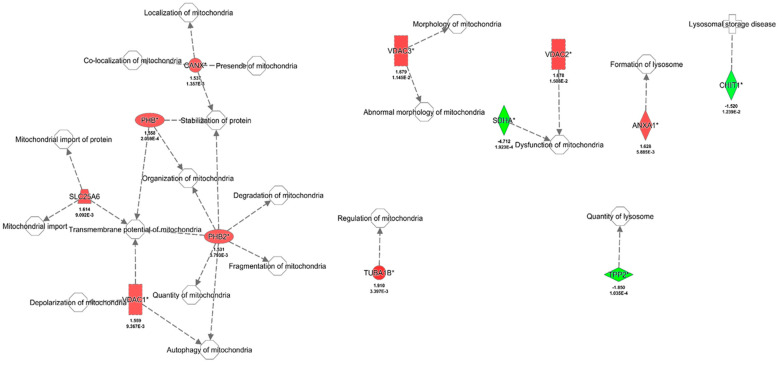
Pathway analysis of HIV+ cocaine vs. HIV+ cocaine and BD1047 in mitochondrial interactions. Significant proteins as determined by fold change (FC) ≤ |1.5| and *p* ≤ 0.05 criteria were identified with an asterisk (*). Significant proteins were corroborated with available literature related to HIV infection. Red indicates upregulated proteins, while green indicates downregulated proteins..

Pretreatment of BD1047 in HIV-1-infected MDM exposed to cocaine uniquely dysregulated eighty (80) proteins, as determined by bioinformatics analyses (Figure 2, Table S2). These proteins have several functions, including cellular remodeling, protein synthesis, cellular migration, endoplasmic reticulum stress, and many other processes (Table 3). However, our interest was to identify dysregulated proteins with a strong relationship with mitochondrial dysfunction, lysosomal damage, CATB networks, and HIV-1 infection. For this reason, IPA analyses were built with networks for these processes. Proteins were selected based on these first networks and needed to have the fold change (FC) ≥ |1.5| and *p* ≤ 0.05 criteria. These relevant proteins were further corroborated and selected based on available and recent literature. Based on the above criteria, for the mitochondrial protein network interactions, twelve (12) proteins were identified (Figure 3). Of those, nine (9) were upregulated: tubulin alpha 1b, two fragments of voltage-dependent ion channel, annexin A1, solute carrier family 25 members, prohibitin, calnexin, and prohibitin 2. The three (3) downregulated proteins in that group included chitinase 1, tripeptidyl peptidase 2, and the succinate dehydrogenase complex. The upregulation of these proteins in mitochondria indicates a serious effect of BD1047 on activating mitochondrial compensation of oxidative respiration proteins (voltage ion channel, solute carrier family 25 members), cytoskeletal remodeling (calnexin, annexin A1, tubulin alpha 1b), and endoplasmic reticulum stress for reducing mitochondrial stress (prohibitin and calnexin) concerning cocaine treatment.

**Figure 4:**
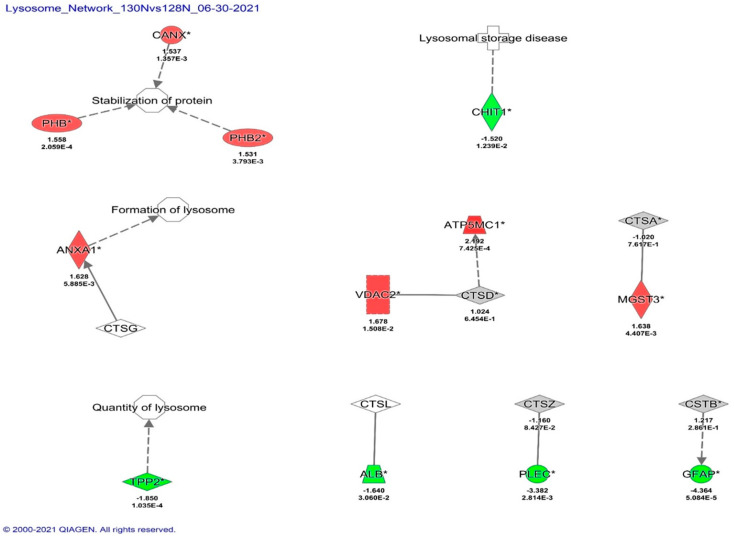
Pathway analysis of HIV+ cocaine vs. HIV+ cocaine and BD1047 affect lysosomal and CATB. Sig-nificant proteins as determined by fold change (FC) ≤ |1.5| and *p* ≤ 0.05 criteria were identified with an asterisk (*). Significant proteins were corroborated with available literature related to HIV infection. Red indicates upregulated proteins, while green indicates downregulated proteins.

The lysosomal and cathepsin network protein interactions, based on IPA analysis, included sixteen (16) dysregulated proteins. Of those, nine (9) were upregulated and seven (7) were downregulated. The upregulated proteins include ATP synthase membrane subunit locus c1, voltage-dependent anion channel 2, microsomal glutathione S-transferase 3, annexin A1, prohibitin, calnexin, prohibitin 2, cystatin B, and cathepsin D. The downregulated proteins include cathepsin A, cathepsin Z, chitinase 1, albumin, tripeptidyl peptidase 2, plectin, and glial fibrillary acidic protein (Table 4, Figure 4). The upregulation of seven proteins in the CATB networks by BD1047 indicates that there are again compensatory mechanisms of the antagonist to reduce oxidative stress within the lysosome/mitochondrial axis (voltage-dependent ion channel 2, microsome glutathione S-transferase and ATP synthase membrane subunit loci c1), an increase of stable lysosomal cytoskeletal integrity (prohibitin, calnexin, prohibitin 2), and regulation of CATB activation by cystatin B and other analogous cathepsin D. Downregulated proteins indicate that other cathepsins and other cytoskeletal proteins are downregulated by BD1047.

**Figure 5:**
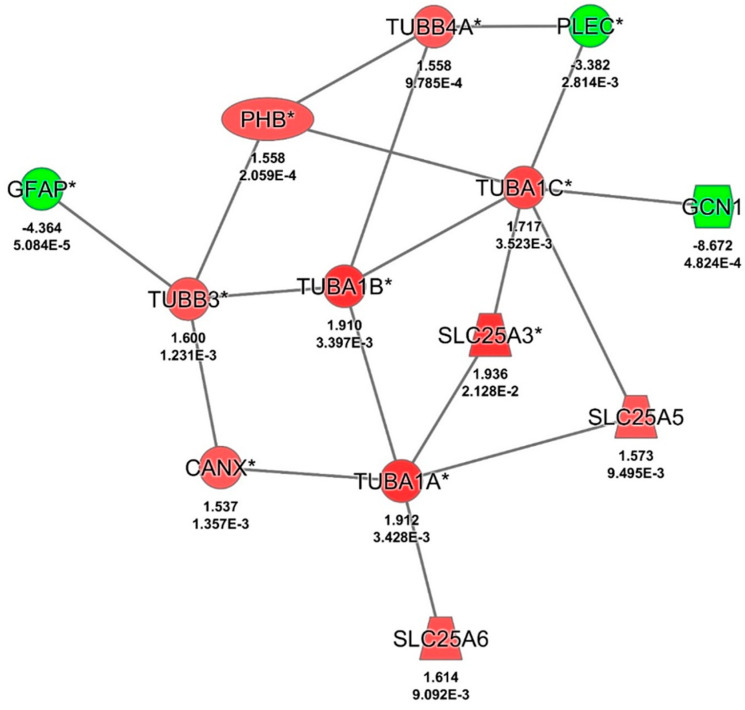
Pathway analysis of HIV+ cocaine vs. HIV+ cocaine and BD1047 in protein interactions. Significant proteins as determined by fold change (FC) ≤ |1.5| and *p* ≤ 0.05 criteria were identified with an asterisk (*). Significant proteins were corroborated with available literature related to HIV infection. Red indicates upregulated proteins, while green indicates downregulated proteins.

Finally, for the HIV-1 IPA protein interaction analyses, fourteen (14) proteins were identified. Of those, ten (10) proteins were upregulated, and four (4) downregulated. The upregulated proteins include solute carrier family 25 member 3, tubulin alpha 1a, tubulin alpha 1b, tubulin alpha 1c, soluble carrier family 25 member 6, tubulin beta 3 class III, solute carrier family 25 member 5, tubulin beta 4A class IVa, prohibitin, and calnexin. Of the downregulated proteins, glial fibrillary acidic protein, plectin, and GCN1 activator of EIF2AK4 were identified (Table 4, Figure 5). Again, the upregulation of these proteins indicates a cellular compensatory mechanism to increase cytoskeletal stability in the membranes that are severely disrupted by the viral cellular takeover. This effect cannot be abrogated by the Sig1R antagonist.

## 4. Discussion

### 4.1. Findings of Differentially Abundant Proteins in HIV-1-Infected MDM Pretreated with BD1047

In this study, we aimed to elucidate the intracellular pathways modulated by BD1047 in HIV-infected macrophages exposed to cocaine. We hypothesized that the Sig1R antagonist BD1047, prior to cocaine exposure, significantly deregulates proteins and pathways involved in HIV-1 replication and CATB secretion that led to neurotoxicity. Differentially abundant proteins in HIV-1 MDM pretreated with BD1047 and exposed to cocaine were subjected to IPA analyses and statistical criteria. However, all the findings were subjected to a recent literature review for further understanding of the underlying mechanisms of infection, mitochondrial dysfunction, and lysosomal/CATB exocytosis. Of these twenty (20) proteins, sixteen (16) are upregulated: ATP synthase membrane subunit c locus 1, ATP synthase C2, ATP synthase C3, ATP synthase D, ATP synthase lipid-binding protein, ADP/ATP translocase, phosphate carrier protein, tubulin alpha chain 1A, tubulin alpha chain 1B, tubulin alpha chain 1C, voltage-dependent anion-selective channel protein 3, outer mitochondrial membrane protein porin 2, Annexin 1, Prohibitin, Calnexin, and transmembrane emp24 domain-containing proteins. Of these, eight (8) proteins are downregulated: four (4) of these proteins were identified by IPA analyses (Surfeit locus protein 4, Obg-like ATPase 1, Plectin, Glial fibrillary acidic protein), and four (4) were selected and added based on previous relevant proteins with HIV + cocaine versus HIV+ groups (Proteasome 26S subunit ubiquitin receptor, non-ATPase 2, UDP-N-acetylhexosamine pyrophosphorylase-like, UDP-N-acetylglucosamine pyrophosphorylase 1 like 1, Stalled ribosome sensor GCN1, glial fibrillary acidic).

It is important to note that BD1047 pretreatment on infected MDM exposed to cocaine has a profound effect on the dysregulation of key proteins. For example, cocaine exposure to infected MDM upregulated one unique protein, the proteasome 26S subunit ubiquitin receptor, non-ATPase 2 (PSMD2) (1.51); BD1047 pretreatment downregulates this protein (−1.42). PSMD2 is a protein complex of the 26S ribosome that binds ubiquitin-damaged proteins, especially viral proteins and lysosomal-damaged proteins [62]. It has been seen that PSMD2 is increased in oxidatively stressed systems or infected cells as a regulatory mechanism to reduce tagged proteins from HIV-1, commonly as an emergency mode. Our results indicate that BD1047 reduces protein dysregulation after viral hijack in these cells.

Other important proteins upregulated by cocaine include: UDP-N-acetylglucosamine pyrophosphorylase 1 like 1 (UAP1L1) (2.22), UDP-N-acetylhexosamine pyrophosphorylase-like (UAP1L1) (2.22), stalled ribosome sensor GCN1 (GCN1) (3.53), and glial fibrillary acidic protein (GFAP) (4.07). These proteins are all downregulated by BD1047 pretreatment in these cells. UAP1L1 is essential in the golgi and endoplasmic reticulum (ER) stress responses to oxidative stress, apoptosis, or viral infections. UAP1L1 is essential for sugar addition to proteins that are going to be degraded or need to be processed for further editing [63]. Results indicate that cocaine increases cellular oxidative stress, and BD1047 abrogates this. Likely, GCN1 is upregulated by cocaine but downregulated after pretreatment with BD1047. GCN1 is a protein related to HIV-1 integration in the nucleus [64] with GCN2, another protein, but also acts as a regulator of proteome quality in stressed or apoptotic cells [65].

Finally, glial fibrillary acidic protein (GFAP) is upregulated by cocaine and downregulated by BD1047 pretreatment. This result is consistent with previous literature on cell models [66]. GFAP is present in MDM and astrocytes and is a hallmark of astrocytosis, which is increased in patients with HIV dementia. Overall, these results indicate that BD1047 uses common (shared) mechanistic proteins on infected MDM exposed to cocaine that reduce ER stress response, proteome translation quality, and cellular structure in an infected and stressed cell model. This could be essential for further therapeutic approaches with this antagonist.

### 4.2. Findings of Unique Differentially Abundant Proteins in HIV-1-Infected MDM Pretreated with BD1047/Cocaine Versus HIV + Cocaine Group

Interestingly, BD1047 pretreatment on infected MDM exposed to cocaine dysregulates eighty proteins. Of those, twenty (20) unique proteins that are not shared with cocaine were selected based on IPA and literature criteria. This indicates that the antagonist activates unique mechanisms for the control of infection, lysosomal damage, mitochondrial dysfunction, and probably CATB exocytosis. The upregulated proteins included ATP synthase F (0) complex subunit C1 (subunits C1-C3 and D), ATP synthase lipid-binding protein (ATP5MC1), ADP/ATP translocase (SLC25A31), phosphate carrier protein (SLC25A3), tubulin alpha chain 1 (subunits 1A-C) (TUB1A-C), voltage-dependent anion-selective channel proteins 1,2,3 (VDAC 1,2,3), Annexin 1 (ANXA1), prohibitin (PHB), calnexin (CANX), and transmembrane emp24 domain-containing protein (TMED10). The downregulated proteins included surfeit locus protein 4 (SURF4), Obg-like ATPase 1 (OLA1), Plectin (PLEC), and glial fibrillary acidic protein (GFAP).

### 4.3. Literature Findings Based on Mitochondrial/Lysosomal/Protein Trafficking

Results indicate that BD1047 treatment uniquely upregulates proteins related to mitochondrial oxidative stress and ATP production, including ATP synthase F(0) complex subunit C1, ATP synthase lipid-binding protein, ADP/ATP translocase 4, and phosphate carrier protein. These proteins are related to counter-regulating mitochondrial stress and increasing ATP synthesis in some cells. This is important to note since the virus readily uses the ATP machinery to obtain its energy from the cell [68]. However, it also increases mechanisms for reducing oxidative stress or promoting apoptosis [69]. ATPase synthase, lipid-binding protein, and phosphate carrier protein promote mitochondrial integrity in many cells by reducing oxidative stress in the cell [70,71].

Other upregulated mitochondrial proteins include voltage-dependent anion-selective channel protein (VDAC 2,3) and Obg-like ATPase (OLA1). Upregulation of proteins such as VDAC 2,3 suggests tight calcium flux control into lysosomes, a major factor for lysosomal permeabilization and cathepsin B leakage. VDAC 2,3 modulates mitochondrial uptake of lysosomal calcium at mitochondria-lysosome contact sites, preventing mitochondrial calcium internalization and permeabilization of lysosomes by calcium. VDAC 1 is the most abundant and interacts with hexokinase and anti-apoptotic proteins, Bcl-2 and Bcl-xL, regulating the traffic of materials through the VDAC1 channel (94).

In terms of protection from endoplasmic reticulum stress, it indicates that there are intracellular mechanisms for counteracting lysosomal dysfunction and cellular trafficking: upregulation of Annexin 1 (ANXA1), prohibitin (PHB), transmembrane emp24 domain-containing protein TMED10, and calnexin (CANX). For example, annexin 1 (ANXA1) (FC = 1.63) and prohibitin (PHB) (FC = 1.56) are related to membrane remodeling for vesicular export and found in endo-lysosomal pathways [112,113]. Furthermore, PHB upregulation decreases lysosomal and mitochondrial dysfunction that might lead to cargo leakage from these organelles, promoting the protection of lysosomes [114]. PHB is essential for mitochondrial damage by modulating oxidative stress [115]. CANX protects against endoplasmic reticulum stress, protein retention, and folding [116]. TEMD10 controls Rab7-LAMP1-positive lysosomes where cathepsin D/B fractions are present for protein and cargo exportation [117]. Transmembrane emp24 domain-containing protein (emp24) indicates that there are intracellular mechanisms for counteracting lysosomal dysfunction and cellular trafficking and promotes vesicular transport among the endoplasmic reticulum and mitochondria [118]. In the same manner, TUB1A interacts with spinster protein on the lysosomal membrane to protect them from leakage [119], and VDAC3 modulates mitochondrial-lysosomal calcium leakage, an essential step for permeabilization [120].

Downregulated proteins participate in cellular trafficking and protective mechanisms against lysosomal and mitochondrial damage. For example, the downregulation of SURF4 reduces the secretion of secretory cargo out of the endoplasmic reticulum and lysosomal leakage [121], and PLEC promotes the cellular trafficking of lysosomal contents and mitochondrial integrity [121]. Low levels of Obg-like ATPase 1 (OLA1) support these findings as well [105,122].

### 4.4. Literature Findings Based on Cathepsin B Exocytosis and Trafficking

We have found in our dataset several highly abundant proteins that might be involved directly or indirectly with pro-CATB and mature cathepsin B trafficking and secretion in our system, including Annexin 1 (ANXA1), prohibitin (PHB), and transmembrane emp24 domain protein (TMED10). While other proteins might be involved in lysosomal disruption and cathepsin leakage, these proteins are of great interest based on a literature review. ANXA1 might be involved in non-classical exocytosis of anti-inflammatory proteins in M2-activated macrophages through the TLR4 receptor, including MMR, CD14, cathepsin B (CATB), CstB, Trx, Anxa1, peptidyl-prolyl cis-trans isomerase A (PPIase), TNF-α, and C-C motif chemokine 2 (CCL2) [123,124]. ANXA1 is mostly related to protein complexes with ANXA2 and ANXA4 for non-classical exocytosis [125]. Prohibitin (PHB) binds to ANXA2, affecting pro-CATB processing [126]. Although not statistically significant in our dataset, ANXA2 binds with ANXA1 and pro-CATB and redirects it to the membrane in several models for exocytosis [126].

Similarly, a high abundance of TMED10 indicates vesicular trafficking of proteins and promotes retrograde transport of exocytotic vesicles and other cargo [127]. Also, TMED10 controls TLR4 signaling with great similarity to ANXA1. In addition, it is found in late endosome Rab7-LAMP1-positive lysosomes where cathepsin D fractions are present for protein and cargo exportation [128]. Rab7-LAMP1 lysosomes might be involved in CATB secretion by ANXA1 or TMED10. Dynamical changes in these proteins or other differentially abundant proteins might affect CATB trafficking and secretion. Functional analyses are needed to prove this. Based on our previous results, BD1047 treatments cause instead of CATB being secreted to be trapped at the membrane into ANXA2/1/TMED10 positive vesicles, caveolae, or CD36 positive vesicles or exosomes, as seen in the literature [129].

**Figure 6:**
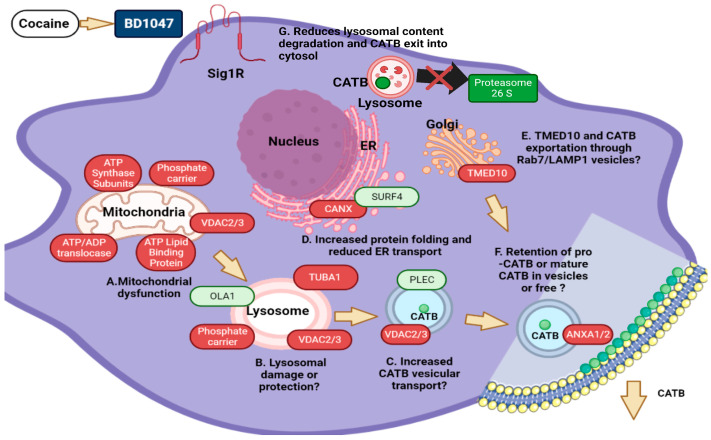
Effects of Sigma-1 receptor agonist BD1047 in preventing HIV infection and cathepsin B release from HIV-1-infected MDM exposed to cocaine. Proposed model on the effect of BD1047 in reversing CATB processing, trafficking, and secretion. Upregulated (red) and downregulated (green). (**A**) Mitochondrial dysfunction, which is evident with the upregulation of selected proteins such as ATP synthase subunits F0 complex (C1–C4 and D), phosphate carrier protein, voltage gate dependent channel proteins (VDAC), ATP/ADP translocase 4, and ATP lipid binding proteins. (**B**) Mitochondrial dysfunction influences lysosomal dysfunction and damage as well. Upregulation of phosphate carrier protein and tubulin 1A (TUBA1) indicates protecting lysosomal integrity. Upregulation of proteins such as VDAC suggests a major factor in lysosomal permeabilization and cathepsin B leakage. Downregulation of Obg-like ATPase protein (OLA1) suggests a protection mechanism against lysosomal leakage. (**C**) Upregulation of VDAC promotes vesicular transport into other organelles. (**D**) Increased protein folding but reduced ER transport. Calnexin (CANX) suggests increased protein folding, processing, and intracellular transport of some proteins, including CATB. Downregulation of surfeit locus protein 4 (SURF4) suggests a concentration of proteins in the ER. (**E**) Upregulation of transmembrane protein emp24 (TMED10) suggests possible movement of Rab7/LAMP1 vesicles for exocytosis. (**F**) Upregulation of ANXA1 suggests a binding of these proteins with pro- and mature CATB either in vesicles or in free form at the cellular membrane or in the cytoplasm. (**G**) BD1047 reduces proteasome activation, indicating that CATB is probably retained in lysosomes and their cargo is not processed through autophagy, where CATB is exposed to the cytosol or fragmented. Model made on BioRender Premium software (https://www.biorender.com/pricing, accessed on 1 August 2024) with available permission for publishing.

The upregulation of PHB also supports this idea. Cell surface CATB has functional significance since this protein can be redistributed from perinuclear lysosomes into peripheral vesicles and associated with plasma membranes, probably in caveolae-rich regions in several cancer cells [130]. We demonstrated that CATB is secreted extracellularly in macrophages [13] and exosomes during HIV-1 infection [131]. A detailed proposed model of the role of deregulated proteins on lysosomal instability, CATB processing, transport, and retention at the membrane of infected MDM can be depicted in Figure 6.

### 4.5. Literature Findings Regarding HIV-1 Infection in MDM

**Figure 7:**
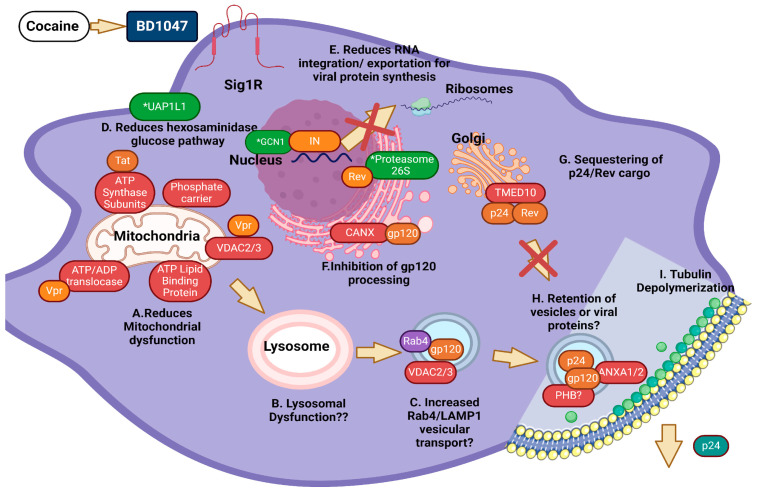
Proposed model on HIV-1 protein processing and viral particle release prevention by BD-1047 on HIV-infected MDM exposed to cocaine. (**A**) Upregulation of ATP synthase F0 complex subunits (C1–C4 and D) indicates a strong oxidative environment indicative of cellular stress. Tat dysregulates this protein. ATP lipid binding protein, phosphate carrier protein, and voltage gate-dependent protein channels 2 and 3 (VDAC2/3) collectively promote mitochondrial permeabilization and damage with several proteins, such as Vpr. (**B**) It is unknown whether mitochondrial damage might promote lysosomal leakage or permeabilization; (**C**) Upregulation of voltage gate-dependent protein channels (VDAC2/3) suggests that vesicular transport increases in Rab4/LAMP1-positive vesicles (two important vesicular proteins) for the exportation of several viral proteins, including gp120. (**D**) There is a reduction in the hexosaminidase pathway for reducing sugar uptake and usage of the virus by UAP1L1. (**E**) Reduction of the mRNA integration by the GCN1 stable sensor with viral integrase and mRNA exportation by PSMD2, which binds to Rev protein. (**F**) Upregulation of calnexin (CANX) suggests inhibition of gp120 processing and transport into other organelles, such as the Golgi apparatus. (**G**) Upregulation of transmembrane emp24 protein (TMED10) suggests possible sequestration of p24 cargo into secretory vesicles. (**H**) Upregulation of prohibitin (PHB) and annexin 1 (ANXA1) might suggest that there is a retention of exocytotic vesicles in infected macrophages. (**I**) HIV-1 p24, gp120, Env, and Gag particles bind to microtubule chains to promote polymerization for HIV-1 vesicle exportation. HIV-1 particles might be retained or incompletely secreted into the extracellular environment. Figure made by BioRender Premium software (1 August 2024) with permission for publishing, *p* ≤ 0.05 criteria were identified with an asterisk (*).

When searching for differentially abundant proteins related to HIV-1 infection, our results demonstrate that BD1047 treatment promotes a dynamic abundance of proteins that have a differential effect on HIV-1 infection in our system. Moreover, BD1047 does not completely reduce the role of cocaine in increasing the abundance of proteins involved in HIV-1 mechanisms of entrance, viral processing, or exportation. Furthermore, BD1047 rather promotes the abundance of certain cellular proteins that might be exploited by the virus for its life cycle while suppressing others. Some of these proteins include ATPase synthase F complex subunits, prohibitin (PHB), and transmembrane protein cargo (emp24). For example, mitochondrial ATPase subunits facilitate HIV-1 transfer in monocytes to CD4-positive cells on the virological synapse [62], while HIV-1 Tat protein increases TUBA1 subunits for effective viral exportation into vesicles [132]. Similarly, prohibitin (PHB) promotes viral exportation into the membrane [89]. Similarly, transmembrane protein cargo (emp24) interacts with HIV-1 Rev protein to export viral particles and endosomal trafficking of infected cells [133]. Upregulation of these proteins indicates that BD1047 treatment does not completely prevent HIV-1 viral entry, processing, and exportation mechanisms in infected MDMs exposed to cocaine. Interestingly, BD1047 treatment also upregulates proteins related to decreased HIV-1 viral entry, movement, and cell replication mechanisms. Some of these proteins include CANX, TMED10, and ANXA1. Therefore, CANX binds to gp120 and inhibits its processing in infected cells [134], while TMED10 limits the retrograde transport of the p24 viral protein inside cells through COPII vesicles [135]. Similarly, ANXA1 limits CCR5 expression in gut PBMCs and is negatively correlated with the simian immunodeficiency virus (SIV) in vitro [86]. In the same manner, low levels of Surf4 indicate that there might be a halt in the replication of certain retroviruses and flaviviruses, including HCV and possibly HIV-1 [104]. These last results appear to be aligned with the reduction of p24 antigen observed after BD1047 treatment in the previous study [1]. Yet, dynamic protein abundance changes might be dependent on other extracellular or intracellular signals, processes, and receptors that need to be fully studied. A proposed model for the effect of BD1047 in downregulation of proteins related to HIV-1 particle release is depicted in Figure 7. 

We are aware of several limitations in our study, including the number of biological samples of healthy women. In the future it will be important to include healthy men. Similarly, we are aware that this study is limited in protein validation through an alternative process such as Western Blot or ELISA. However, we believe that our downstream process is robust enough with high statistical criteria that it can be readily used for further studies.

## 5. Conclusions

Complementary therapies are required to reduce the neurotoxic effects of HIV-1 infection and cocaine in the brain. Infected MDMs migrate into the brain synergistically with drugs of abuse and secrete several factors that promote neuroinflammation and toxicity. One of those factors is the lysosomal-derived protein CATB. In a previous study, we demonstrated that the Sig1R antagonist BD1047 at 10 µM reduced CATB secretion and HIV-1 levels when added one hour before cocaine exposure in vitro [1]. These results highlight the importance of this antagonist as a potential therapy as well as the importance of studying intracellular signaling to understand the mechanisms of action in MDMs.

In the present study, we have quantified the proteome from HIV-infected macrophages treated with the Sig1R antagonist BD1047 prior to cocaine exposure. We aimed to understand the intracellular pathways activated and associated proteins modified by BD1047 in HIV-1-infected MDM exposed to cocaine. We wanted to examine their role in HIV infection, lysosomal and mitochondrial dysfunction, and CATB trafficking and secretion. For this purpose, we applied TMT quantitative proteomics to MDM lysates of HIV-infected, cocaine-exposed, BD1047-treated, and their respective controls [1]. Our results indicate that BD1047 pretreatment reduces key proteins that are shared with the HIV + cocaine group regarding protein synthesis, folding, and protein control (Proteasome 26S subunit ubiquitin receptor, non-ATPase 2, UDP-N-acetylhexosamine pyrophosphorylase-like, UDP-N-acetylglucosamine pyrophosphorylase 1 like 1, Stalled ribosome sensor GCN1, glial fibrillary acidic). These proteins are downregulated by BD1047 pretreatment, indicating that the antagonist abrogates cocaine action on proteome dysregulation mechanisms.

However, BD1047 also dysregulates eighty (*n* = 80) proteins when compared with the HIV+ cocaine group. Of those twenty (*n* = 20), proteins were selected based on IPA analyses and a literature review. Our findings indicate that BD1047 activates counterregulatory mechanisms for reducing mitochondrial permeability, dysfunction, and lysosomal permeability. Also, dysregulated proteins indicate that the antagonist might modulate protein trafficking and exportation of infected MDM through several proteins and mechanisms. However, these mechanisms might not completely describe the role of BD1047 in controlling CATB trafficking and exocytosis. CATB exocytosis can be done by several processes that include lysosomal permeabilization and secretion. We are aware that key proteins such as ANXA1, PHB, and CANX are involved in CATB exocytosis in other biological models, as evidenced in the literature.

Similarly, quantitative proteomic evidence demonstrates that the antagonist can reduce certain viral processes within the MDM. However, as seen in our previous study [1], BD1047 significantly reduces HIV-1 p24 antigen levels when compared with infected MDM exposed to cocaine. Although many proteins have dissimilar effects on different parts of the viral cycle, proteins such as ANXA1, PHB, and TMED10 play a key role in HIV-1 entry and exportation. Other proteins are essential for this process as well. However, understanding of the role of BD1047 pretreatment on infected MDM exposed to cocaine needs to be further analyzed for deeper understanding and possible use as a potential therapeutic approach in HIV-1 patients who are cocaine abusers.

## Figures and Tables

**Table 1:** Dysregulated proteins and functions in HIV+ vs. HIV− MDM.

Uniprot ID	Name	Gene ID	Subcellular Localization	Associated Pathway	Fold Change	*p*-Value
E9PMV1	Plectin	PLEC	Cytosol/Cytoskeleton	Actin-Binding	2.7	6.7 × 10^−3^
H0Y8X1	Succinate dehydrogenase complex flavoprotein subunit A	SDHA	Mitochondrial Complexes	Mitochondrial Electron Transport Chain	3.5	4.0 × 10^−4^
A0A0B4J213	Large ribosomal subunit protein eL30	RPL30	Ribosomes/cytosol/Rough ER	Protein Synthesis	−1.9	5.8 × 10^−3^
A0A0C4DH44	Large ribosomal subunit protein eL30	RPL30	Ribosomes/cytosol/Rough ER	Protein Synthesis	−1.9	5.8 × 10^−3^

Red fold change (FC) = upregulated proteins (FC ≥ |1.5| and *p*-value ≤ 0.05) and green FC = downregulated proteins (FC ≥ |1.5| and *p*-value ≤ 0.05).

**Table 2:** Upregulated proteins between HIV+ cocaine vs. HIV+ MDM.

Uniprot ID	Name	Gene ID	Subcellular Localization	Associated Pathway	Fold Change	*p*-Value
* H7C1H2	Proteasome 26S subunit ubiquitin receptor, non-ATPase 2	PSMD2	Cytosol	Protein regulation/ Degradation	1.5	1.91 × 10^−3^
A0A087X226	UDP-N-acetylglucosamine pyrophosphorylase 1 like 1	UAP1L1	Cytosol/ Intermediate filaments	Growth intermediate filaments	2.2	7.7 × 10^−4^
Q3KQV9	UDP-N-acetyl hexosamine pyrophosphorylase-like	UAP1L1	Cytosol/Intermediate Filaments	Growth/intermediate filaments	2.2	7.7 × 10^−4^
Q92616	Stalled ribosome sensor GCN1	GCN1	Cytosol/ Ribosomes	Control of ribosomal activity	3.5	4.7 × 10^−4^
A0A1W2PQU7	Glial fibrillary acidic protein fragment	GFAP	Intermediate filaments	Cell morphology	4.0	1.9 × 10^−5^
A0A1W2PS58	Glial fibrillary acidic protein fragment	GFAP	Intermediate filaments	Cell morphology	4.1	1.9 × 10^−5^
K7EPI4	Glial fibrillary acidic protein fragment	GFAP	Intermediate filaments	Cell morphology	4.1	1.9 × 10^−5^

Asterisk *—Only one protein is unique among the group of comparisons of HIV+ cocaine versus HIV+. Six (6) other proteins are unique among the comparisons. Upregulated proteins (FC ≥ |1.5| and *p*-value ≤ 0.05).

**Table 3:** Dysregulated proteins and functions in HIV-positive cocaine vs. HIV-positive cocaine and BD1047-treated MDM.

Name	FC	*p*-Value	Function by Literature Review	
			HIV-1	Lysosome/Mitochondrial Dysfunction
ATP synthase F (0) complex subunit C1, mitochondrial	2.2	7.0 × 10^−3^	Facilitates HIV-1 transfer in monocytes to CD4-positive cells [62]. Tat 101 protein reduces the expression of ATP synthase in Jurkat cells and promotes mitochondrial dysfunction [63].	Accumulates in lysosomes of neurons with Batten’s disease lysosomal disorders. contains all the machinery proteins for vesicular fusion and phagosome formation [64]. Formation of the transition pore complex that promotes mitochondrial degradation [65,66].
ATP synthase lipid-binding protein	2.2	7.0 × 10^−3^	No information.	Present in isolates of cells with proteins in lysosomes of Batten’s disease [67,68].
ADP/ATP translocase 4	2.0	8.6 × 10^−3^	Binds to HIV-1 Vpr protein and promotes mitochondrial membrane permeabilization [69,70,71].	Promotes mitochondrial permeabilization and storage of cargo protein in associated lysosomes [72].
Phosphate carrier protein, mitochondrial	1.9	2.1 × 10^−2^	Regulates mitochondrial permeabilization [73].	Integral mitochondrial protein [74].
Tubulin Alpha Chain 1A- C subunits	1.9	3.4 × 10^−3^	Bind to microtubule chains to promote polymerization for HIV-1 vesicle exportation [75,76,77].	Reduced TUB1A (tubulin alpha chain subunits) related to impaired lysosomal cargo in neurites in vitro and in vivo [78]. Interacts with spinster protein on the lysosomal membrane to protect lysosomes from leakage [79].
Voltage-dependent anion-selective channel protein 3 (VDAC3)	1.7	2.1 × 10^−2^	Vpr induces T cell and Jurkat cell apoptosis and mitochondrial permeabilization and reduces its expression through the transition pore complex [80,81].	Prevents mitochondrial calcium internalization and permeabilization of lysosomes by calcium [80,81].
Outer mitocondrial membrane protein porin 2 (fragment) (VDAC2)	1.7	1.5 × 10^−2^	Modulates apoptosis and membrane permeabilization. Its expression is inhibited by HIV-1 [69]	Regulates Ca+2 channel to prevent lysosome leakage of cathepsins and autophagy [80,81].
Annexin 1 (fragment)	1.6	5.9 × 10^−2^	ANXA 2 interacts with Gag in CD63 compartments for virion exportation into cells [82]. ANXA2 binds to serine protease inhibitors for viral entry into macrophages [83].	Coupled in tetramer at the cell surface for excretion and retention of cathepsins [84]. Transduction of Tat-ANXA1 inhibits cyclooxygenase and promotes an anti-inflammatory response in raw 264.7 cells [85] ANXA1 signaling is dysfunctional in SIV infection and may contribute to chronic inflammation [86]. ANXA1 is correlated with exosomes of CATB protein in P2XR-activated cells [87,88]
Prohibitin	1.6	2.0 × 10^−4^	Prohibitin 1/2 heterodimer interacts with HIV-1 glycoprotein for viral spread [89].	Reduces mitochondrial UPR response [90] Modulates oxidative stress and mitochondrial dysfunction [91]. Downregulation of mitochondrial PHB is a crucial event in mitochondrial damage [92]
Voltage-dependent anion-selective channel protein VDAC-1	1.6	9.4 × 10^−3^	Its expression is inhibited by HIV-1 [69,82]. HIV-1Tat dysregulates VDAC-1 inducing ATP release and cell death [93].	Allows for communication between the mitochondrion and the cell mediating the balance between cell metabolism and cell death [94].
Calnexin (fragment)	1.5	1.3 × 10^−3^	HIV-Nef modulates calnexin, suppressing cholesterol flux [95]. High Binding of calnexin to gp120 promotes inefficient gp120 processing [96]. Nef associated with Calnexin promotes lipid accumulation in the endoplasmic reticulum (ER) and binds to gp120 [97].	Involved with proteins destined for secretion, endosomal reticulum stress. Chaperone that protects and retains protein secretion [98].
Transmembrane emp24 domain-containing protein	1.5	1.2 × 10^−2^	Rev interacts with the protein for the secretion of viral particles [99].	Vesicular trafficking of proteins promotes retrograde transport of exocytotic vesicles and other cargo [100]. Controls TLR4 signaling. Found in late endosome Rab7-LAMP1-positive lysosomes where cathepsin D fractions are present for protein and cargo exportation [101].
Proteasome 26S Subunit Ubiquitin Receptor	−1.4	1.4 × 10^−3^	PSMD2 interacts with Vpr for viral infectivity [102,103]	Ubiquitin of proteins that are damaged [102].
Surfeit locus protein 4	−1.6	1.1 × 10^−3^	Regulates and promotes replication of HCV in replication complexes and other positive-strand viruses [104].	Maintenance of the architecture of the endoplasmic reticulum (Golgi). Cargo protein of secretory proteins out of ER [104].
Obg-likeATPase 1 (Fragment)	−1.8	3.8 × 10^−3^	Interacts with p17 and promotes CD4 T cell proliferation and autophagy inhibition [105].	Hydrolyzes ATP and can also hydrolyze GTP with lower efficiency [105].
UDP-N-acetyl-hexosamine phosphorylase type	−3.0	2.0 × 10^−5^	Helps with immune function of IF3 and activation against viral infection [106].	Adds glucosamine and other sugars to the proteins being transported in ER and other cargo [106].
UDP-N-acetyl-glucosamine pyrophosphorilase type	−3.0	2.0 × 10^−5^	Helps with the immune function and activation against viral infection [59,106].	Adds sugars to protein being transported in ER and other cargo [106].
Plectin (Fragment)	−2.13	9.0 × 10^−3^	CXCR4 signaling is related to the modulation of autophagy [52].	Pectin-stabilized actin filaments aids in the autophagosome–lysosome fusion that supports autophagy [107].
Glial fibrillary acidic protein (Fragment)	−4.4	5.1 × 10^−5^	Increases its expression in HIV+-infected macrophages and astrocytes after ER stress [108]	Cytoskeletal fragments of astrocytes and occasionally in monocytes [109]
Stalled ribosome sensor GCN1	−8.7	4.8 × 10^−5^	Combines with GCN2 for HIV-1 integration into nucleus [110]	Reduces protein synthesis if translation is not correct [111]

Red fold change (FC) = upregulated proteins (FC ≥ |1.5| and *p*-value ≤ 0.05), and green FC = downregulated proteins (FC ≥ |1.5| and *p*-value ≤ 0.05). Red = upregulated proteins (FC ≥ |1.5| and *p*-value ≤ 0.05), and green = downregulated proteins (FC ≥ |1.5| and *p*-value ≤ 0.05).

**Table 4:** Description of selected differentially abundant proteins in HIV-infected MDM exposed to cocaine after BD-1047 treatment versus HIV + cocaine groups selected after IPA and literature analyses. Fold change: red = upregulated proteins, green = downregulated proteins.

Uniprot ID	Name	Gene ID	Subcellular Localization	Associated Pathway	FC	*p*-Value
D6R9H7	ATP synthase F subunit C1	ATP5MC1	Mitochondrial Membrane	Mitochondrial ATP Formation	2.2	7.0 × 10^−3^
Q06055	ATP synthase C2	ATP5MC2	Mitochondrial Membrane	Mitochondrial ATP Formation	2.2	7.0 × 10^−3^
P48201	ATP synthase C3	ATP5MC3	Mitochondrial Membrane	Mitochondrial ATP Formation	2.2	7.0 × 10^−3^
O75947	ATP synthase D	ATP5PD	Mitochondrial Membrane	Mitochondrial ATP Formation	2.2	7.0 × 10^−3^
E7EPU7	ATP synthase lipid-binding protein	ATP5MC1	Mitochondrial Membrane	Mitochondrial ATP Formation	2.2	7.0 × 10^−3^
Q9H0C2	ADP/ATP translocase 4	SLC25A31	Mitochondrial Membrane	Mitochondrial ATP/ADP Formation	2.0	8.6 × 10^−3^
F8VWQ0	Phosphate carrier protein, mitochondrial	SLC25A3	Mitochondria	Transporter of phosphate ions	1.9	2.1 × 10^−2^
F8W0F6	Tubulin Alpha Chain 1A subunit	TUBA1A	Cytoskeleton	Microtubule Formation	1.9	3.4 × 10^−3^
F8VRK0	Tubulin Alpha Chain 1B subunit	TUBA1B	Cytoskeleton	Microtubule Formation	1.9	2.1 × 10^−2^
F8VS66	Tubulin Alpha Chain 1C subunit	TUBA1C	Cytoskeleton	Microtubule Formation	1.7	3.5 × 10-3
E5RFP6	Voltage-dependent anion-selective channel protein 3 (VDAC 3)	VDAC3	Mitochondrion Outer membrane	Transporter of anions into mitochondria	1.7	1.1 × 10^−3^
A2A3S1	Outer mitocondrial membrane protein porin 2 (Fragment) (VDAC2)	VDAC2	Mitochondrion Outer Membrane	Mitochondrial Transport	1.7	1.5 × 10^−2^
P21796	Outer mitocondrial membrane protein porin 2 (Fragment) (VDAC1)	VDAC1	Mitochondrion Outer Membrane	Mitochondrial Transport Mediate balance between metabolism and cell death	1.6	9.3 × 10^−3^
Q5N3T0	Annexin 1 (fragment)	ANXA1	Endosomes, apical, basolateral membrane, extracelular exosome, nucleus, cillium, phagocytic cup	Exocytosis of calcium activated proteins	1.6	5.9 × 10^−2^
P35232	Prohibitin	PHB	Mitochondrion inner membrane, nucleus, cell membrane, cytoplasm	Maintains protein integrity and mitochondrial integrity	1.6	2.0 × 10^−4^
D6RAQ8	Calnexin (fragment)	CANX	Endoplasmic reticulum	Chaperone Secretory Pathway ER	1.5	1.3 × 10^3^
P49755	Transmembrane emp24 domain-containing protein	TMED10	Golgi apparatus, cis-Golgi, trans-Golgi, endoplasmic reticulum, cell membrane, secretory vesicle, melanosome	Early secretory pathway between COPI and COPII vesicles	1.5	1.2 × 10^−2^
H7C1H2	Proteasome 26S Subunit Ubiquitin Receptor non-ATPase 2	PSMD2	Cytoplasm	Protein regulation and degradation	−1.4	1.4 × 10^−3^
B7Z1G8	Surfeit Locus Protein 4	SURF4	Membrane	Endoplasmic reticulum/Golgi cargo to membrane	−1.6	1.0 × 10^−3^
C9JTK6	Obg-like ATPase (fragment)	OLA1	Centrosome/Cytosol	Hydrolyzes ATP and GTP	−1.8	4.0 × 10^−3^
Q3KQV9	UDP-N-acetylhexosamine pyrophosphorylase- like	UAP1L1	Endoplasmic reticulum/cargo	Add sugars to cargo for degradation	−3.0	2.1 × 10^−5^
A0A087X226	UDP-N-acetylglucosamine pyrophosphorylase- like-1	UAP1L1	Endoplasmic reticulum/cargo	Add sugars to cargo for degradation	−3.0	2.1 × 10^−5^
E9PKG0	Plectin (fragment)	PLEC	Cytoskeleton	Interlinks microtubules with filaments	−3.3	3.0 × 10^−3^
A0A1W2PQU7	Glial Fibrillary Acidic Protein (GFAP)	GFAP	Intermediate filaments	Cytoskeleton of many cells including astrocytes	−4.3	5.1 × 10^−5^
Q9261	Stalled Ribosome Sensor GCN1	GCN1	Ribosomes/Cytosol	Controls protein synthesis by inhibiting certain factors	−8.7	4.8 × 10^−4^

## Data Availability

Most of the data generated in this study are included in this manuscript. The proteomics raw data sets have been deposited in the PrteomeXChange [136] consortium via de PRIDE repository [137] and is readily available with a dataset identifier: Project accession PXD052318 DOI: 10.6019. Alternatively, the reviewer can access the dataset by logging into the PRIDE website using the following account details: Username: reviewer_pxd052318@ebi.ac.uk, Password: DwgAITT58tRL.

## Supplementary Materials

The following supporting information can be downloaded at: https://www.mdpi.com/article/10.3390/biomedicines12091934/s1, Figure S1: SDS PAGE for MS/MS.tiff; Table S1: TMT Tags Description; Table S2: Dysregulated Proteins in HIV infection, cocaine exposure, and BD1047 treatment.

## Gender Quality in Our Research

We fully support gender equality in our research. Our MDMs are taken from the blood PBMCs of healthy women. No male donors were added. The main reason for using female donors includes our previous research involving women cohorts. We estimate that HIV-1 infection and CATB secretion might differ among men and women. Yet, this effect has not been proven.
