# Supplementary material for: Analysis of Sigma-1 Receptor Antagonist BD1047 Effect on Upregulating Proteins in HIV-1-Infected Macrophages Exposed to Cocaine Using Quantitative Proteomics
Source: Biomedicines. 2024 Aug 23;12(9):1934. doi: 10.3390/biomedicines12091934 (PMC11428496; doi:10.3390/biomedicines12091934)
Supplement: Supplementary file 1 [file biomedicines-12-01934-s001.zip › Table S1 TMT Tags Description.pdf]

TMT Label	Infection Status	Treatment
126	HIV-	Non-treated
127N	HIV+	Non-treated
127C	HIV-	Cocaine
128N	HIV+	Cocaine
128C	HIV-	Sig1R Antagonist (BD1047)
129N	HIV+	Sig1R Antagonist (BD1047)
129C	HIV-	BD1047+cocaine
130N	HIV+	BD1047+cocaine
130C	*Internal Control – Pool of controls*	

Table S1. TMT Tags Description. An example of one TMT10-plex kit sample distribution used to run each of the 8 samples + pool of controls from all donors. A total of 24 samples were run in three TMT kits.
