# Supplementary figures and images for: Analysis of Sigma-1 Receptor Antagonist BD1047 Effect on Upregulating Proteins in HIV-1-Infected Macrophages Exposed to Cocaine Using Quantitative Proteomics
Source: Biomedicines. 2024 Aug 23;12(9):1934. doi: 10.3390/biomedicines12091934 (PMC11428496; doi:10.3390/biomedicines12091934)

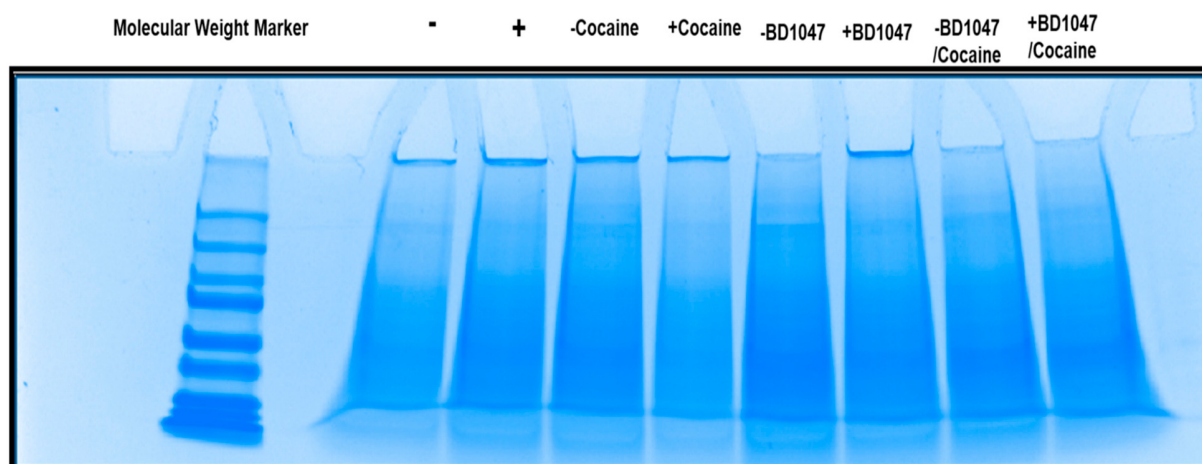

Figure S1: SDS PAGE for MS/MS.tiff;

Supplement: Supplementary file 1 [file biomedicines-12-01934-s001.zip › Figure S1.pdf]
